# The Effects of Berry Extracts on Oxidative Stress in Cultured Cardiomyocytes and Microglial Cells: A Potential Cardioprotective and Neuroprotective Mechanism

**DOI:** 10.3390/molecules27092789

**Published:** 2022-04-27

**Authors:** Tanisha L. Currie, Marguerite M. Engler, Cara H. Olsen, Victor Krauthamer, Jonathan M. Scott, Patricia A. Deuster, Thomas P. Flagg

**Affiliations:** 1 Graduate School of Nursing, Uniformed Services University of the Health Sciences, Bethesda, MD 20814, USA; marguerite.engler@usuhs.edu; 2 Department of Preventive Medicine and Biostatistics, Uniformed Services University of the Health Sciences, Bethesda, MD 20814, USA; cara.olsen@usuhs.edu; 3 Department of Biomedical Engineering, George Washington University, Washington, DC 20052, USA; vkrauthamer@email.gwu.edu; 4 Department of Military and Emergency Medicine, Uniformed Services University of the Health Sciences, Bethesda, MD 20814, USA; jonathan.scott@usuhs.edu (J.M.S.); patricia.deuster@usuhs.edu (P.A.D.); 5 Department of Anatomy, Physiology, and Genetics, Uniformed Services University of the Health Sciences, Bethesda, MD 20814, USA

**Keywords:** antioxidants, anthocyanin, cyanidin-3-glucoside, delphinidin-3-rutinoside, black currant, cranberry

## Abstract

Oxidative stress is a key underlying factor in cognitive decline and atherosclerosis. Oxidative stress occurs at the cellular level with an imbalance between reactive oxygen species and reactive nitrogen species and a deficiency in antioxidants. Mounting evidence suggests that berry flavonoids may promote cellular health by exerting antioxidant properties. Black currant and various berry extracts were tested in microglia (BV-2) and cardiomyocyte (HL-1) cell lines to study their biological effects. The principal ingredients in black currant and cranberry extract–delphinidin 3-rutinoside (D3R) and cyanidin 3-glucoside (C3G), were also assessed. A menadione-induced oxidative stressor was used, and its output was quantified to detect oxidative stress (CellROX^TM^). Black currant extract had similar antioxidant effects as N-acetylcysteine (NAC) in HL-1 cells with regard to cellular protection, whereas cranberry extract was ineffective. In contrast, cranberry extract was comparable in effectiveness to black currant extract in BV-2 cells. D3R and C3G also reduced oxidative stress similarly to whole berry extracts, which indicates that these ingredients may confer the antioxidant effects of berries. Black currant and cranberry extracts inhibit oxidative stress in microglial and cardiomyocyte cell lines. Black currant extract was more effective in reducing oxidative stress in the HL-1 cells, whereas cranberry extract was comparable in reducing oxidative stress in the BV-2 cells. The results suggest that berry flavonoids exert neuro- and cardioprotective effects.

## 1. Introduction

Optimal brain and cardiovascular health (CV) are paramount for an individual’s overall health and well-being. Oxidative stress due to an imbalance of reactive oxygen and nitrogen species or a deficiency of antioxidants in the body is an important contributor to brain and CV health [1]. Reactive oxygen species (ROS) were first discovered in phagocytes as part of the defense response, but excessive ROS can have detrimental biological effects. For instance, production of the potent vasodilator, nitric oxide (NO), is markedly reduced during prolonged oxidative stress which can lead to endothelial dysfunction and CVD [1,2,3]. A reduction in NO production and an increase in inflammatory cytokines may result in the inhibition of neurotransmitters such as serotonin, thereby leading to depression and microglial activation [4,5]. Therefore, developing strategies to inhibit oxidative stress may be a viable strategy to improve brain and CV health. 

Dietary supplementation with whole berries or berry extracts may promote brain and CV health as an alternative to pharmacologic interventions. Epidemiological and interventional studies have shown that flavonoids found in fruits and vegetables offer neuroprotective and CV benefits [6,7]. Common berries and foods containing flavonoids are black currants, chocolate, tea, wine, grapes, cherries, cranberries, and blueberries [8]. Flavonoids are plant-derived, polyphenolic compounds with anti-inflammatory, neuroprotective, cardioprotective, and antioxidative properties [6,9,10,11,12]. Some common subclasses of bioactive flavonoids include anthocyanins and proanthocyanidins [PACs] [13,14]. Anthocyanins are the plant pigments with a basic, chemical structure composed of flavylium cations which can be linked to different sugars such as glucose, rhamnose or xylose [15,16]. Anthocyanins are water-soluble, and contribute to the rich color sources of red, purple, and blue naturally characterized in fruits, flowers, and plants depending upon their pH levels [15,17,18]. Anthocyanin content in different fruits and vegetables varies. However, the total anthocyanin content of berries such as black currant and cranberry is generally greater than 150 mg/100 g [14,17,18,19]. Common anthocyanins found in black currants include delphinidin-3-glucoside, delphinidin 3-rutinoside, cyanidine-3-glucoside, and cyanidine-3-rutinoside, which have been associated with decreasing blood pressure, improving lipid profiles, and promoting brain health [8,10,20,21,22,23,24]. The second class of bioactive compounds, PACs, are oligomers of flavan-3-ol with leucoanthocyanidins as precursors in their biosynthetic pathway [25,26]. PACs are naturally occurring polyphenols widely distributed in plants and their leaves [13,26]. They are found in red wine, grapes, and other berries such as cranberry, elderberry and aronia berries. They provide an antimicrobial defense against herbivores, and bacteriostatic effect for maintenance of urinary tract health in humans [13,26]. Therefore, black currants, were of particular interest in the current investigation due to their high anthocyanin content and potential health benefits [20,21].

Presently, there are significant gaps in the literature on the effect of black currants and other berry extracts on brain and CV health at the cellular level. Although some researchers have explored the effects and composition of certain types of flavonoids (e.g., those founds in vegetables, chocolate, legumes and spices such as curcumins), few studies have addressed the role of berry flavonoids in reducing oxidative stress on cardiomyocyte and microglial cells. Specifically, our investigation examined how black currant extract may play a role in reducing oxidative stress at the cellular level. We investigated the effects of black currant extract on HL-1 cardiomyocytes derived from the atrial cardiac muscle of a transgenic mouse [27] and BV-2 microglial cells derived from mouse bone marrow [28,29]. The aim of the present study was to compare the effects of black currant with other berry extracts to determine whether they equally inhibit the production of induced oxidative stress. 

## 2. Results

### 2.1. Effects of Berry Extracts on Oxidative Stress within HL-1 Cells

**Figure 1:**
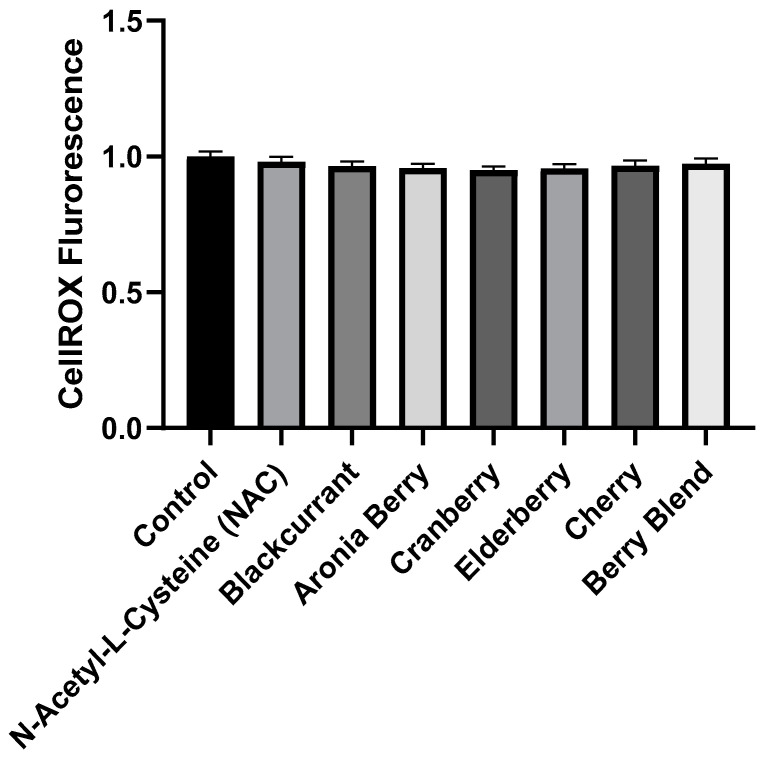
Effects of berry extracts (1 µg/mL) on oxidative stress in HL-1 cells. Mean (+ specify if SEM or SD) normalized CellROX^TM^ fluorescence from experiments (*n* = 3) demonstrating the effect of berry extracts on basal oxidative stress in HL-1 cardiomyocytes. In each experiment, CellROX^TM^ fluorescence was normalized to the control. There were no significant effects (*p* > 0.05) of NAC or any of the berry extracts on CellROX^TM^ fluorescence in the absence of an oxidative stress-inducing agent.

To investigate the potential effects of black currant flavonoids on oxidative stress, concentrated black currant extract was obtained from Artemis International, Inc. (Ft Wayne, IN, USA). For comparison, we also obtained extracts from aronia berry, cranberry, elderberry, cherry and berry blend (composition of mixed berries as mentioned above). The powdered extracts were dissolved in deionized water at a concentration of 100 mg/mL and further diluted into culture media for each experiment. HL-1 cardiomyocytes were first treated for 1 h with black currant or other berry extracts (1 µg/mL) to determine if they had any effect on basal oxidative stress. As a control, cells were also treated with a potent antioxidant, N-acetyl cysteine (NAC), at a concentration of 1 mmol/L. Oxidative stress was assessed using CellROX^TM^ as described in the Methods. As shown in Figure 1, neither NAC nor any of the berry extracts affected CellROX^TM^ fluorescence consistent with the conclusion that there is little or no oxidative stress in HL-1 cardiomyocytes in the absence of a stressor.

**Figure 2:**
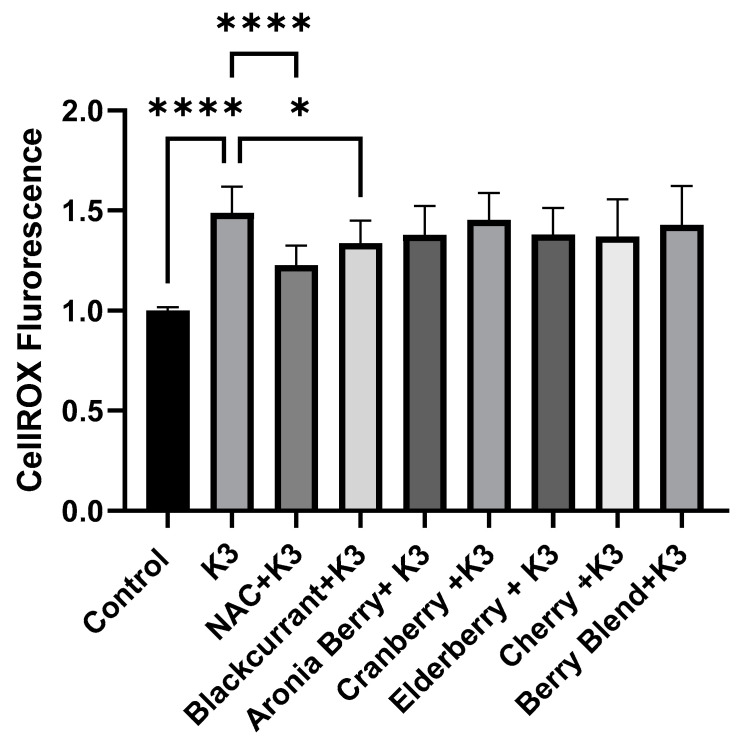
Effects of berry extracts (1 µg/mL) on K3-induced oxidative stress in HL-1 cells. Mean normalized CellROX^TM^ fluorescence from experiments (*n* = 3) demonstrating the effect of berry extracts on menadione (Vitamin K3) induced oxidative stress in HL-1 cells. In each experiment, CellROX^TM^ fluorescence was normalized to the Control. K3 (100 µM) elicited a significant increase in CellROX^TM^ fluorescence (**** *p* < 0.0001) indicating a marked increase in oxidative stress. The potent antioxidant N-acetylcysteine (NAC, 1 mM) significantly reduced K3-induced oxidative stress (**** *p* < 0.0001). Among the berry extracts, only black currant extract markedly inhibited oxidative stress (* *p* < 0.05).

Next, the ability of the berry extracts to counteract the oxidative stress induced by menadione (also known as vitamin K3) was determined. As shown in Figure 2, K3 induced a marked increase in CellROX^TM^ fluorescence. As expected, pretreatment with the potent antioxidant, NAC, significantly inhibited K3-induced oxidative stress (*p* < 0.001). Among the berry extracts, only black currant extract significantly reduced oxidative stress induced by K3 (*p* = 0.037). In contrast, none of the other berry extracts inhibited K3-induced oxidative stress. For example, oxidative stress in the presence of K3 + cranberry extract was nearly identical to treatment with K3 alone. Based on these results, black currant and cranberry extracts were selected for further investigation to better understand the differences between the radical scavenging activity of these particular berry extracts and their components. 

### 2.2. Effects of Black Currant and Cranberry Extracts on HL-1 Cell Viability 

**Figure 3:**
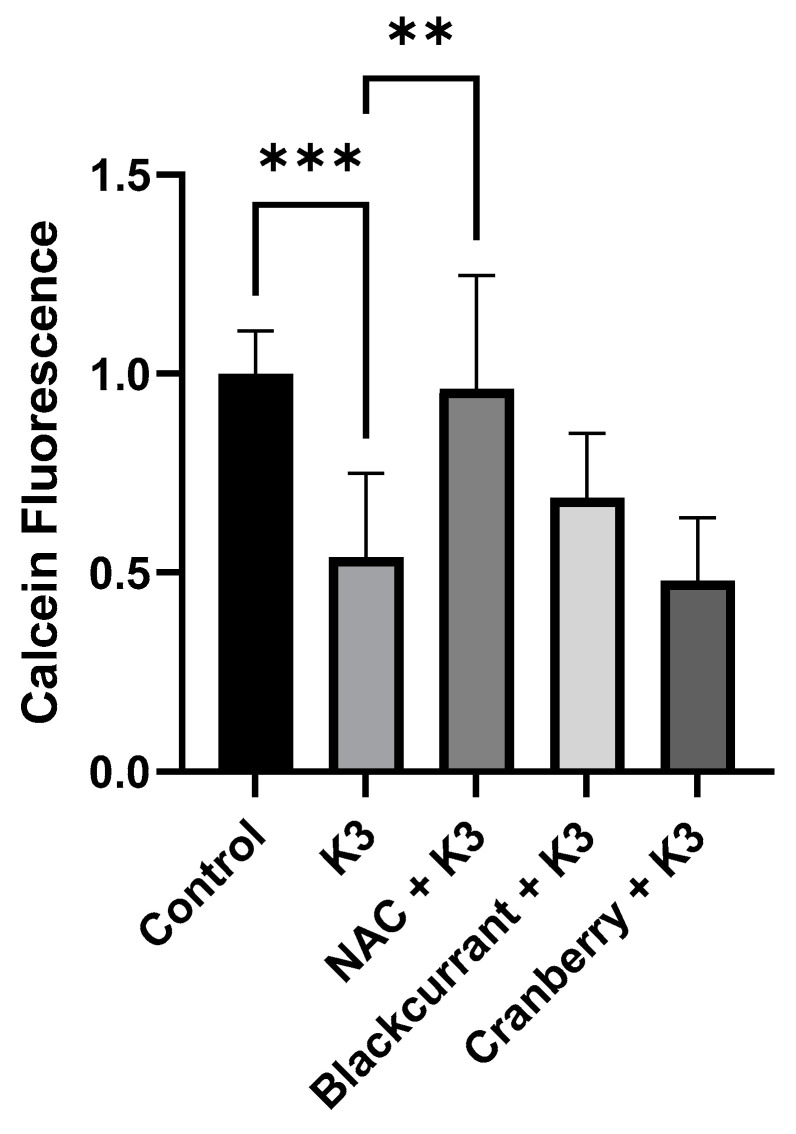
Effects of berry extracts on cell viability in HL-1 cells. Shown are normalized results of calcein-AM staining of live cells following treatments as indicated. As expected, K3 induced a significant reduction in cell viability (*** *p* < 0.001), whereas 1 mM NAC significantly inhibited K3-induced cell death (** *p* < 0.01). Although neither berry extract significantly affected cell viability, black currant extract (100 µg/mL) offered modest protection when compared to cranberry extract (100 µg/mL) which offered no protection from cell death caused by K3.

Increases in oxidative stress are associated with increased cell death. Therefore, cell viability with HL-1 cardiomyocytes was determined. The results showed that K3 induced a significant increase in cell death as assessed by calcein-AM staining as described in the methods. As shown in Figure 3, treatment with K3 induced significant cell death (*p* < 0.001), whereas NAC inhibited K3-induced cell death (*p* < 0.01). Interestingly, neither black currant nor cranberry extracts (100 µg/mL) exhibited significant effects on cell viability. However, it was apparent that black currant extract caused a modest improvement in viability, whereas cranberry extract appeared to have no effect, in agreement with the results observed for oxidative stress measurement. Taken together, these results suggest that chemical differences in the makeup of different berry extracts is associated with berry-specific abilities to inhibit oxidative stress and cell survival; however, it may also reflect the cell type being tested. Therefore, we examined the effects of black currant and cranberry extracts on BV-2 microglial cells.

### 2.3. Effects of Black Currant and Cranberry Extracts on Oxidative Stress and Cell Viability in BV-2 Cells

**Figure 4:**
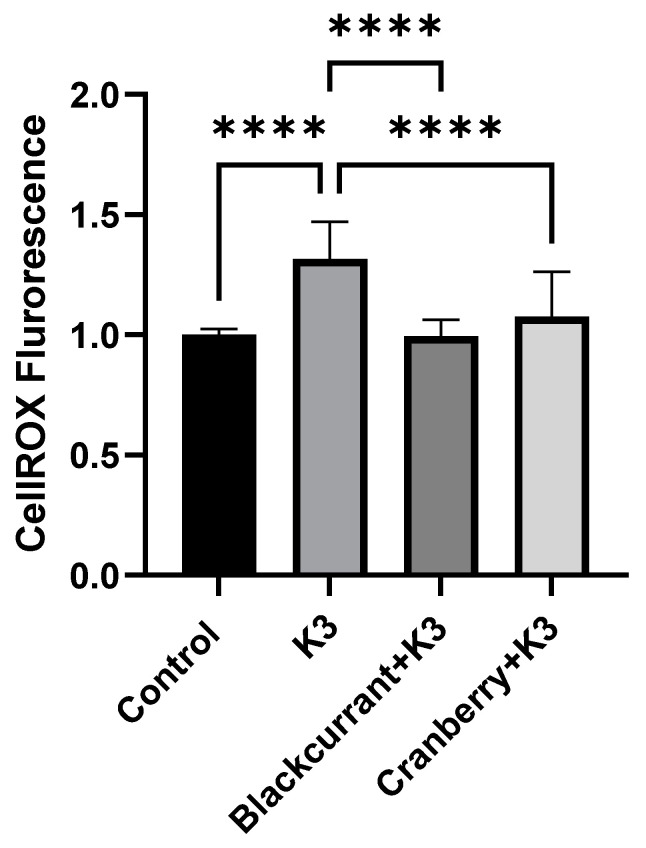
Effects of berry extracts on oxidative stress in BV-2 cells. Mean normalized CellROX^TM^ fluorescence from experiments (*n* = 12) demonstrating the effect of berry extracts on menadione (Vitamin K3) induced oxidative stress in BV-2 cells. In each experiment, CellROX^TM^ fluorescence was normalized to the Control. K3 (100 µM) elicited a significant increase in CellROX^TM^ fluorescence (**** *p* < 0.0001) indicating a marked increase in oxidative stress. Both cranberry (100 µg/mL) and black currant extract (100 µg/mL) inhibited oxidative stress induced by menadione (**** *p* < 0.0001).

In HL-1 cells, black currant extract appeared to be more effective at inhibiting oxidative stress than cranberry extract. Neither extract affected basal oxidative stress in BV-2 cells (*data not shown*) as expected. In contrast to the results using HL-1 cells, however, both black currant and cranberry extracts (100 µg/mL) were similarly effective in reducing K3-induced oxidative stress (Figure 4). 

### 2.4. Effects of Black Currant and Cranberry Extracts on Cell Viability in BV-2 Cells

**Figure 5:**
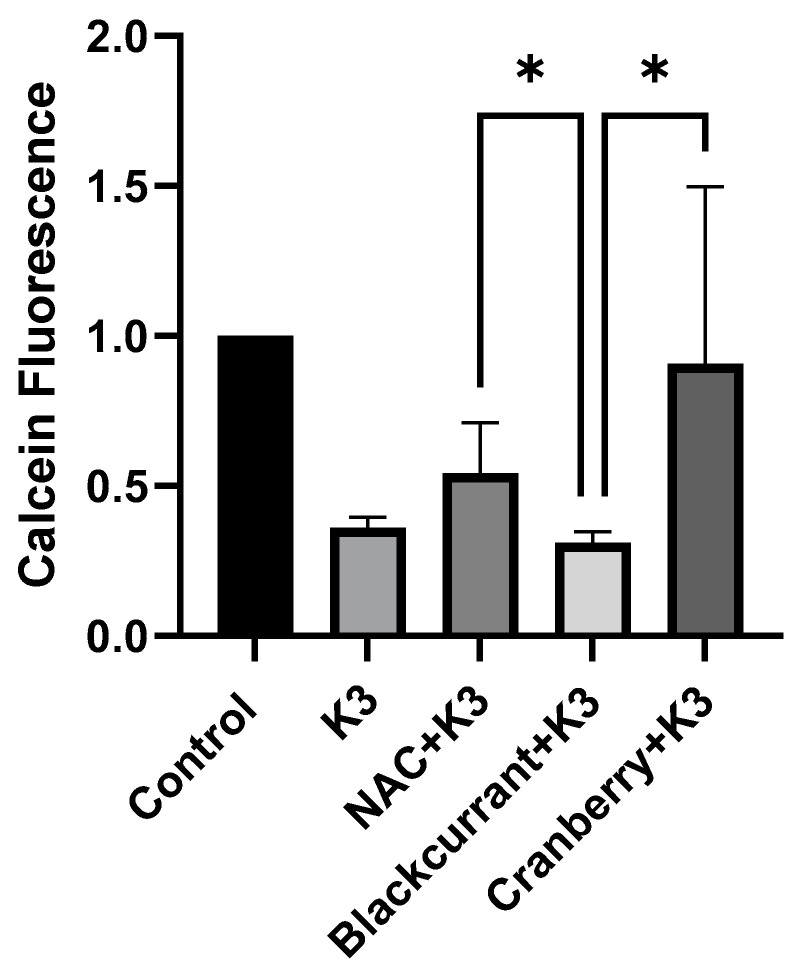
Effects of berry extracts (100 µg/mL) on cell viability in BV-2 cells. This figure demonstrates menadione (K3)-induced oxidative stress which decreased cell viability in comparison to the nontreatment group. As for treatment groups, cranberry extract+K3 and black currant (100 µg/mL) + K3 were nonsignificant for cell viability compared to K3 only. Although cranberry extract + K3 (100 µg/mL) did not show significance in cell viability compared to NAC+K3, it had the highest average cell viability among the other treatment groups. Due to the large standard deviation in one group, group means were compared by Welch’s ANOVA followed by Dunnett’s pairwise comparisons, and the only significant differences were between NAC and black currants (100 µg/mL) (* *p* = 0.0451) and between cranberry (100 µg/mL) and black currants (100 µg/mL) (* *p* = 0.0178).

We also compared the effects of the berry extracts on cell viability (Figure 5). As in the HL-1 cells, treatment with K3 was associated with significant cell death as assessed by calcein-AM fluorescence. Because both extracts were equally effective at suppressing oxidative stress, we predicted that both extracts would be effective at inhibiting K3-induced cell death. In contrast to the results in HL-1 cells, neither black currant extract (100 µg/mL) nor the potent antioxidant, NAC (1 mmol/L), provided a significant protective benefit. This suggests that inhibition of oxidative stress is not sufficient to prevent K3-induced cell death in BV-2 cells. Interestingly, cranberry extract (100 µg/mL) was modestly successful in preserving cell viability in the presence of K3. This result suggests that K3 may induce cell death through mechanisms that do not require oxidative stress or that BV-2 cells may be more sensitive to oxidative damage that even potent antioxidants such as NAC are unable to suppress. The observation that cranberry extract provides modest survival benefit suggests that there may be other benefits to berry extracts that remain to be explored. 

### 2.5. Effect of Delphinidin-3 Rutinoside (D3R) and Cyanidin-3-Glucoside (C3G) on Oxidative Stress in BV-2 Cells

**Figure 6:**
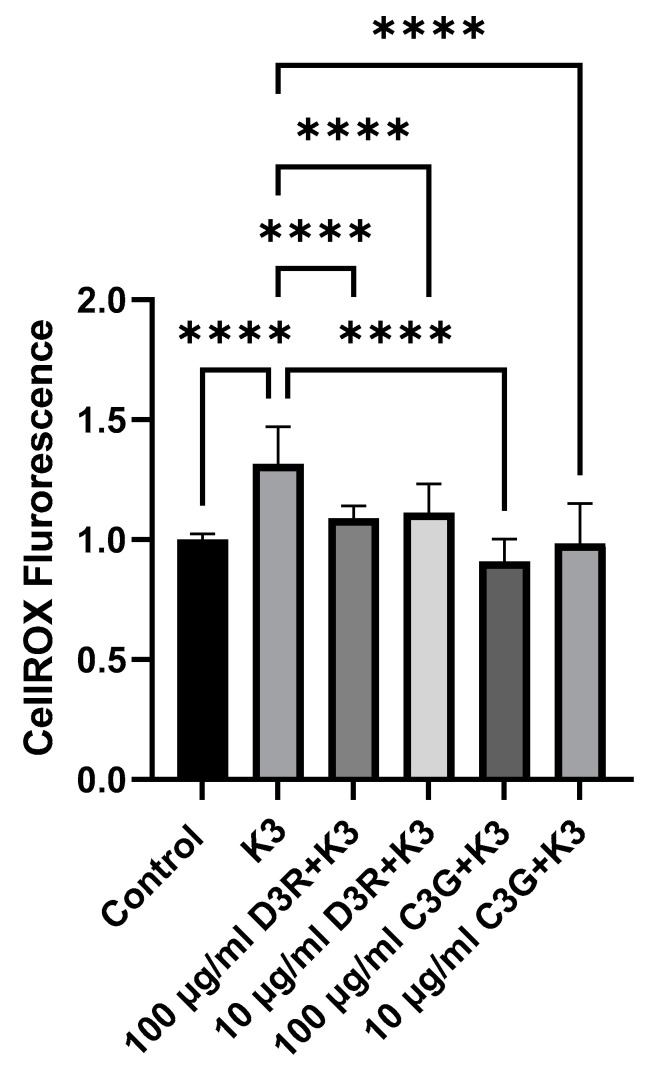
Effects of anthocyanins on oxidative stress in BV-2 cells. Shown are results of CellROX^TM^ fluorescence from experiments (*n* = 3) demonstrating the effect of two different anthocyanins, D3R and C3G, on K3-induced oxidative stress in BV2 cells. Both D3R and C3G inhibited K3-induced oxidative stress in BV-2 cells at concentrations of 100 μg/mL and 10 μg/mL (**** *p* < 0.0001).

There are many anthocyanins at varying concentrations in different berry extracts; however, delphinidin-3-rutinoside (D3R; 304.91 mg/100 g) and cyanidin-3-glucoside (C3G; 25.07 mg/100 g) are major anthocyanins found in black currants [17,18,19]. Therefore, we investigated the effects of isolated anthocyanins on oxidative stress induced by K3 (Figure 6). Both C3G and D3R at two different dose concentrations (100 μg/mL and 10 μg/mL) exhibited antioxidant effects and inhibited K3-induced oxidative stress. This result is consistent with the conclusion that the antioxidant activity of the individual anthocyanins underlies the antioxidant effect of black currant extract. 

## 3. Discussion 

Oxidative stress is a key mechanism linked to endothelial dysfunction, decreased NO and promotion of inflammatory cytokines such as tumor necrosis factor-α and interleukin factors, all of which can lead to pathophysiological processes of atherosclerosis in heart disease and neurological conditions [1,30]. Oxidative stress and its downstream pathology have been a long-standing issue of human physiology. Flavonoids such as those found in berries could potentially benefit human health through their reported properties of inhibition of oxidative stress, regulation of NO and neuro- and cardioprotective mechanisms [7,11,30,31]. In this study, berry extracts were investigated in a systematic approach in specific cell models (HL-1 and BV-2 cells) to determine their potential effects and roles in oxidative stress. By understanding these cellular effects, specific nutritional interventions, dose concentrations, and targeted treatment can be further developed and advanced to help mitigate oxidative stress contributing to chronic conditions of neurological and cardiovascular diseases. 

### 3.1. Comparing the Effects of Berry Extracts on Oxidative Stress in HL-1 and BV-2 Cells

Black currant extract was more effective than cranberry extract at reducing K3-induced oxidative stress in HL-1 cells. In BV-2 cells, however, the antioxidant capacity of black currant and cranberry extracts were similar. This surprising finding suggests that not all cell types respond to the extracts in the same way. It is not clear why such a difference should exist, but it may reflect different transport processes in the cell membrane that modulate flavonoid uptake and accumulation. Although the transport processes for flavonoids have not been clearly defined, members of the ATP-binding cassette (ABC) family of transporters are likely involved and may be differentially expressed in different cells [32]. Alternatively, it can be speculated that the origin of oxidative stress in the two cell types is different, which leads to varying levels of effectiveness for the berry extracts. 

### 3.2. Role of Antioxidant Activity in Promoting Cell Viability in HL-1 and BV-2 Cells

The data show that black currant extract is capable of attenuating oxidative stress in HL-1 cardiomyocytes and that this correlates with improved cell viability. N-acetylcysteine (NAC), a potent antioxidant, was used as a control in cell viability assays. In a previous study pretreating with NAC as an antioxidant under H_2_O_2_-induced oxidative stress condition, significantly reduced oxidative stress and increased glutathione levels in H9C2 cardiomyocyte cells [33]. This is consistent with the observations in the present study where NAC was most effective at reducing menadione-induced oxidative stress and preserving cell viability in HL-1 cells. The observation that black currant was the most effective at inhibiting oxidative stress and preserving HL-1 cell viability suggests that both NAC and black currant may exploit similar cellular antioxidant pathways to promote cell survival. 

In parallel experiments using BV-2 cells, markedly different results were observed. Interestingly, neither NAC nor black currant extract was effective at preserving cell viability. While our results did not reach statistical significance for cranberry extract, likely as a result of the large confidence intervals, the cranberry extract exhibited higher mean cell viability than black currant in BV-2 cells exposed to K3. This suggests that the antioxidant properties of NAC and black currant extract may not be contributing to cell survival in BV-2 cells in the same way that they do in HL-1 cells. Previous studies have shown that cranberry extracts enriched in anthocyanins improve BV-2 cell viability consistent with our results [34]. Cranberry extract contains other bioactive flavonoids including quercetin and proanthocyanidins (PAC) [13,22,35,36,37,38]. A known benefit of PACs is their antiadherence and antimicrobial properties, which inhibit urinary tract infections, inflammation and periodontal bacteria [26,37,38]. Further studies are needed to explore the biochemical basis of the different effects of the extracts on different cell types.

### 3.3. Comparison of Extract Components on Oxidative Stress on BV-2 Cells

Black currant extract was effective at reducing oxidative stress in the present study, in agreement with previous studies of other fruit extracts such as tart cherry, grape seed, lingonberry and American ginseng berry [37,38,39,40,41]. Black currant is a robust of anthocyanins [14,17,18,19].Consistent with the hypothesis that anthocyanins are a major contributor to the antioxidant activity of the black currant extract, both delphinidin-3-rutinoside (D3R) and cyanadin-3-glucoside (C3G) significantly suppressed menadione-induced oxidative stress in BV-2 cells. D3R and C3G have been associated with both cardioprotective and neuroprotective benefits. D3R, in particular, possesses antioxidant properties, reduces intracellular ROS and provides bovine ciliary smooth muscle relaxation [42,43]. C3G has been found to suppress ROS and protect endothelial vascular cells from inflammatory-related damage. It has also been associated with gastroprotective and antiplatelet properties [22,44]. Understanding the beneficial properties of the key dominant active compounds as well as the whole berry extract effects on cell models provide important information and may be applicable for further human studies targeting individual health. 

### 3.4. Summary

Taken together, the present study provides evidence supporting the conclusion that black currant and cranberry extracts harbor antioxidant activity that may exert cardioprotective and neuroprotective effects. The high levels of anthocyanins in the extracts likely contribute to the antioxidant properties of the extracts. Furthermore, the results indicate that improvements in cell viability may not be solely dependent on the antioxidant activity and suggest that there may be other biologically active compounds (e.g., proanthocyanidins) that promote cell survival in other ways. Furthermore, while these studies focused on oxidative stress and cell viability in HL-1 and BV-2 cells, we cannot rule out other systemic effects of berry extracts that may be important for promoting human health. For example, proanthocyanidins have been associated with glucose lowering and hypocholesterolemic activities [13]. 

## 4. Materials and Methods

### 4.1. Cell Culture

The effects of berry extracts and their major component compounds on cell survival and ROS were examined in two different cell types—HL-1 cardiomyocytes and BV-2 microglial cells. HL-1 cells were maintained in supplemented Claycomb medium at 37 °C in a humidified incubator with 5% CO_2_. Supplemented Claycomb medium (Sigma) contains fetal bovine serum (10% *v*/*v*), penicillin/streptomycin (1000 U/mL), norepinephrine (0.1 mM), and l-Glutamine (2mM). Cells were grown in T75 flasks coated with gelatin and fibronectin until 90% confluency and then passaged into 96 well plates for treatments and experiments. Cells were typically sub-cultured twice a week using 0.05% Trypsin/EDTA for dissociation. BV-2 cells were maintained at 37 °C in a humidified incubator under 5% CO_2_ in BV2 media consisting of DMEM supplemented with fetal bovine serum (10% *v*/*v*) and penicillin/streptomycin (10 mL) and DMEM. Cells were grown in T75 and T150 flasks until 90% confluency and then split before treatments and experiments. Cells were subcultured once a week using 0.25% Trypsin/EDTA, BV2 microglia media, and Phosphate Buffered Saline (PBS) for cell wash. Cells were plated on 6 well or 96 well plates for experiments. 

### 4.2. Berry Extracts

Concentrated berry extracts from the following berries: black currant, aronia berry, cranberry, elderberry, cherry, and berry blend (composition of mixed berries as mentioned above) were donated in powder form by Artemis International, Inc. (Ft Wayne, IN, USA). A Certificate of Analysis was provided for berry extracts to ensure the product’s content and purity. Dried berry extracts were dissolved in water (100 mg/mL) and stored in the freezer at −20 °C until experimentation. Berry extracts were added to the culture media for the cell being tested at specific concentrations as noted for each experiment. 

In a subset of experiments, the effectiveness of major components of the berry extracts—including delphinidin-3-rutinoside (Sigma, cat# PHL80735, St. Louis, MO, USA) and cyanidin 3-glucoside (Sigma, cat# PHL89616, St. Louis, MO, USA) was examined. These compounds were used in parallel experiments with berry extracts to assess the relative antioxidant effects that might be attributable to each.

### 4.3. Cell Viability Assay

To determine the toxicity of the various berry extracts, the Cell Health Assay Kit (CellSignal Technology; Cat # 13837, Danvers, MA, USA) was used. Briefly, cells were plated in a 96 well plate and incubated with berry extracts at 100 µg/mL in cell culture media for 1 h. Calcein-AM fluorescent dye was added to the cells at a concentration of 1 µM to label living cells for 30 min. The membrane-permeant calcein-AM dye was rapidly de-esterified in living cells with active esterase. The Cell Health Assay Kit was able to detect at a range from 500–50,000 cells per well. Calcein fluorescence elicited a wavelength of 485 nm and was measured at 520 nm by using a microplate fluorescence reader.

### 4.4. Oxidative Stress Assay

To assess cellular oxidative stress, the CellROX^TM^ Green Assay (Invitrogen, cat. # C10444, Waltham, MA, USA) was employed following the manufacturer’s protocols. Briefly, cells were plated in a 96 well plate and incubated with menadione (vitamin K_3_; Cayman Chemical, cat # 15950) to induce oxidative stress in the presence or absence of berry extracts at different concentrations as noted in the text. At the conclusion of the treatment, CellROX^TM^ dye was added to the cells for 30 min. Following washing with PBS, resultant fluorescence elicited a wavelength of 485 nm and was measured at 520 nm using a microplate fluorescence reader. In all experiments, a canonical antioxidant (1 mM *N*-acetylcysteine) was used as a positive control to ensure the quality of the results.

### 4.5. Data Analysis

Statistical analysis was performed using Microsoft Excel (version 10) and Graph Pad (version nine) software. Data were analyzed for both the primary and secondary aims using ANOVA and *t*-tests comparing the differences between the means of the berry and control groups. The primary outcome was induced oxidative stress. Tukey’s HSD or Dunnett’s tests were performed as appropriate if overall significance was observed. The results were considered statistically significant if the *p* level was <0.05. There were no privacy issues with data considering cells have no sensitive or identifying data. Data were primarily stored and managed in Microsoft Excel. To ensure experiment validity, a control was put in place such as a known potent antioxidant, *N*-acetylcysteine, to compare with the berry extracts. Additionally, experiments were repeated no less than three times to ensure data integrity. Data were expressed as mean with the standard deviation. 

## 5. Conclusions

In conclusion, the results of this study demonstrated that black currant and cranberry extracts exert antioxidant properties that may benefit human health. Black currant extract effectively inhibited ROS in HL-1 cardiomyocytes and protected them from cell death. In BV-2 cells, the anthocyanins, C3G and D3R, were both as effective in their role on oxidative stress as the berry extracts themselves. Taken together, the results indicate that the effects of the extracts are not equal, and there is some cell specificity to the response, which is a novel finding that was not anticipated.
